# Architectures and accuracy of artificial neural network for disease classification from omics data

**DOI:** 10.1186/s12864-019-5546-z

**Published:** 2019-03-04

**Authors:** Hui Yu, David C. Samuels, Ying-yong Zhao, Yan Guo

**Affiliations:** 1 Department of Internal Medicine, University of New Mexico, Albuquerque, NM 87131 USA; 2 Vanderbilt Genetics Institute, Department of Molecular Physiology and Biophysics, Vanderbilt University Medical School, Nashville, TN 37232 USA; 3 Key Laboratory of Resource Biology and Biotechnology in Western China, School of Life Sciences, Northwest University, Xi’an, 710069 Shaanxi China

**Keywords:** Supervised classification, Cancer diagnosis, Artificial neural network, Deep learning, Omics

## Abstract

**Background:**

Deep learning has made tremendous successes in numerous artificial intelligence applications and is unsurprisingly penetrating into various biomedical domains. High-throughput omics data in the form of molecular profile matrices, such as transcriptomes and metabolomes, have long existed as a valuable resource for facilitating diagnosis of patient statuses/stages. It is timely imperative to compare deep learning neural networks against classical machine learning methods in the setting of matrix-formed omics data in terms of classification accuracy and robustness.

**Results:**

Using 37 high throughput omics datasets, covering transcriptomes and metabolomes, we evaluated the classification power of deep learning compared to traditional machine learning methods. Representative deep learning methods, Multi-Layer Perceptrons (MLP) and Convolutional Neural Networks (CNN), were deployed and explored in seeking optimal architectures for the best classification performance. Together with five classical supervised classification methods (Linear Discriminant Analysis, Multinomial Logistic Regression, Naïve Bayes, Random Forest, Support Vector Machine), MLP and CNN were comparatively tested on the 37 datasets to predict disease stages or to discriminate diseased samples from normal samples. MLPs achieved the highest overall accuracy among all methods tested. More thorough analyses revealed that single hidden layer MLPs with ample hidden units outperformed deeper MLPs. Furthermore, MLP was one of the most robust methods against imbalanced class composition and inaccurate class labels.

**Conclusion:**

Our results concluded that shallow MLPs (of one or two hidden layers) with ample hidden neurons are sufficient to achieve superior and robust classification performance in exploiting numerical matrix-formed omics data for diagnosis purpose. Specific observations regarding optimal network width, class imbalance tolerance, and inaccurate labeling tolerance will inform future improvement of neural network applications on functional genomics data.

**Electronic supplementary material:**

The online version of this article (10.1186/s12864-019-5546-z) contains supplementary material, which is available to authorized users.

## Background

In the past decade, deep neural networks have inspired waves of novel applications for machine learning problems. Recently, the biomedical field has also witnessed a surge of deep learning assisted studies, which involve protein structure prediction, gene expression regulation, protein classification, etc. [1]. For instance, in just 3 years, a series of deep learning models [2–5] was devised to map DNA/RNA sequence motifs and boost transcription factor binding estimation. In addition, deep learning models have been built for classifying metagenomics [6] and predicting heart failure [7], suicide risk [8], hospital re-admission [9], and disease outcomes [10].

Many deep learning applications use feedforward artificial neural network models [11]. Perceptrons [12] are the simplest form of feedforward neural networks which consist of only two layers (input and output). Multi-Layer Perceptrons (MLPs) extend from perceptrons by embedding one or more hidden layers. MLP and alike models had a long and continual record of successes in the supervised classification of high-throughput molecular data. One of the best well-known examples is the classification of four subtypes of small-round-blue-cell tumors, executed on 63 training subjects and 25 testing subjects in 2001 [13]. This pioneering study applied a Linear Perceptron, a two-layered neural network with a linear activation function. Following this seminal study, artificial neural network models gained great popularity in the supervised classification of microarray expression data [14–16]. Very recently, a two-layered artificial neural network was adapted to achieve excellent prognosis prediction of the new generation of gene expression profiles, RNA-Seq data [17].

Convolutional Neural Network (CNN) is a recent divergent variant of MLP, comprising one or more convolutional layers followed by one or more fully connected layers. With demonstrated advantage in processing images and videos, CNN becomes a trendy jackknife for various machine learning applications, and the biomedical domain is no exception. A notable application of CNN in biological studies is DeepBind, which predicts the sequence specificities for hundreds of DNA- and RNA-binding proteins [2]. Most recently, CNN was applied to mine medical records for predicting hospital re-admission [9]. More adapted CNN models for biomedical research are on the horizon [18, 19].

Decades ago, it was proposed that one hidden layer with an appropriate number of neurons (units) suffices the “universal approximation” property [20, 21]. It is generally agreed that a neural network with more than one hidden layer can be regarded as a deep architecture, so CNNs and MLPs of two or more hidden layers are classified as deep learning model. With neural network models re-gaining popularity in the deep learning wave, it is worthwhile to interrogate the additional merit brought forth by the “deeper” architecture particularly. As a matter of fact, doubtful voices arose. For example, despite its name, DeepBind may not necessarily be “deep,” because many of its models are composed of merely one convolution step along with its associated operations. A subsequent survey of DeepBind and related CNN architectures concluded that deep architectures are not necessary for the motif discovery task [22]. In another evaluative work, it was suggested that the deep learning approaches may not be suitable for metagenomic classification [23].

Nevertheless, deep architectures are rapidly emerging for tackling the disease diagnosis problem. One study [24] leveraged an unsupervised deep learning method, the sparse autoencoder, to transform feature representation before a traditional supervised model (Logistic Regression) was employed for disease classification. Only minor improvement was brought forth by the sparse autoencoder [24]. Another study [25] applied a CNN-rooted Generative Adversarial Network to discriminate cancer samples from normal samples using microarray data. This study was implemented on only two datasets, and the results were compared between the Generative Adversarial Network and two baselines based on Restricted Boltzmann Machines. Without comparing to a wide panel of alternative models and without testing on a good number of datasets, the alleged advantage of the proposed deep learning model was not convincing. These recent trials did not successfully prove deep neural networks as a superior choice in exploiting omics data for diagnosis purpose. It is under-addressed whether deep neural network architectures is as promising for this purpose as in other successful applications [1]. Explicit and unambiguous guidance is expected to inform the data scientists and computational researchers in the community at large.

Given the uncertain performance in omics-based anomaly classification yet demonstrated successes in other biomedical research sectors, deep neural network models deserve a thorough evaluation in the setting of well-structured genomics datasets, being benchmarked against their shallow analogs as well as classical models beyond neural networks. Herein, we evaluated the performance of MLP and CNN relative to classical machine learning models for disease classification on omics data (including high throughput transcriptome and metabolome datasets). Observations and conclusions made in this study are informative for researchers who are interested in applying deep learning techniques to predict anomaly status from omics data.

## Results

### Single-layered MLPs with ample hidden units perform better than deeper MLPs

**Fig. 1:**
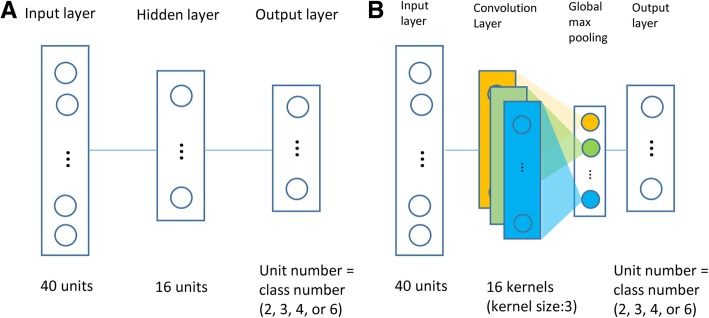
The basic architectures for MLP (**A**) and CNN (**B**). Because MLP’s and CNN’s basic architectures both had a single hidden/convolution layer of 16 units or kernels, they were both coded as “1L_16U.” Starting from 1L_16U, variant architectures with increasing number of units on hidden layers and/or additional hidden layers were included into the testing panel (Additional file 1: Table S1). While not shown in the plot, the architectures by default have a dropout layer immediately prior to the output layer with a dropout rate of 0.5

We first compared the relative performances of MLP/CNN across six primary architectures (Additional file 1: Table S1) of varying depths (number of layers) and widths (number of units at a layer), which derived from the basic structures (Fig. 1) inspired by a related evaluation study [22]. In total, 37 dataset-specific performance values, in ACC or Kappa, are generated for each architecture. Within each dataset, we converted the original performance values for six models to ranks, and evaluated the overall performance of each architecture by the average rank across 37 datasets. The architecture with minimal average rank was regarded as the overall best structure.

**Fig. 2:**
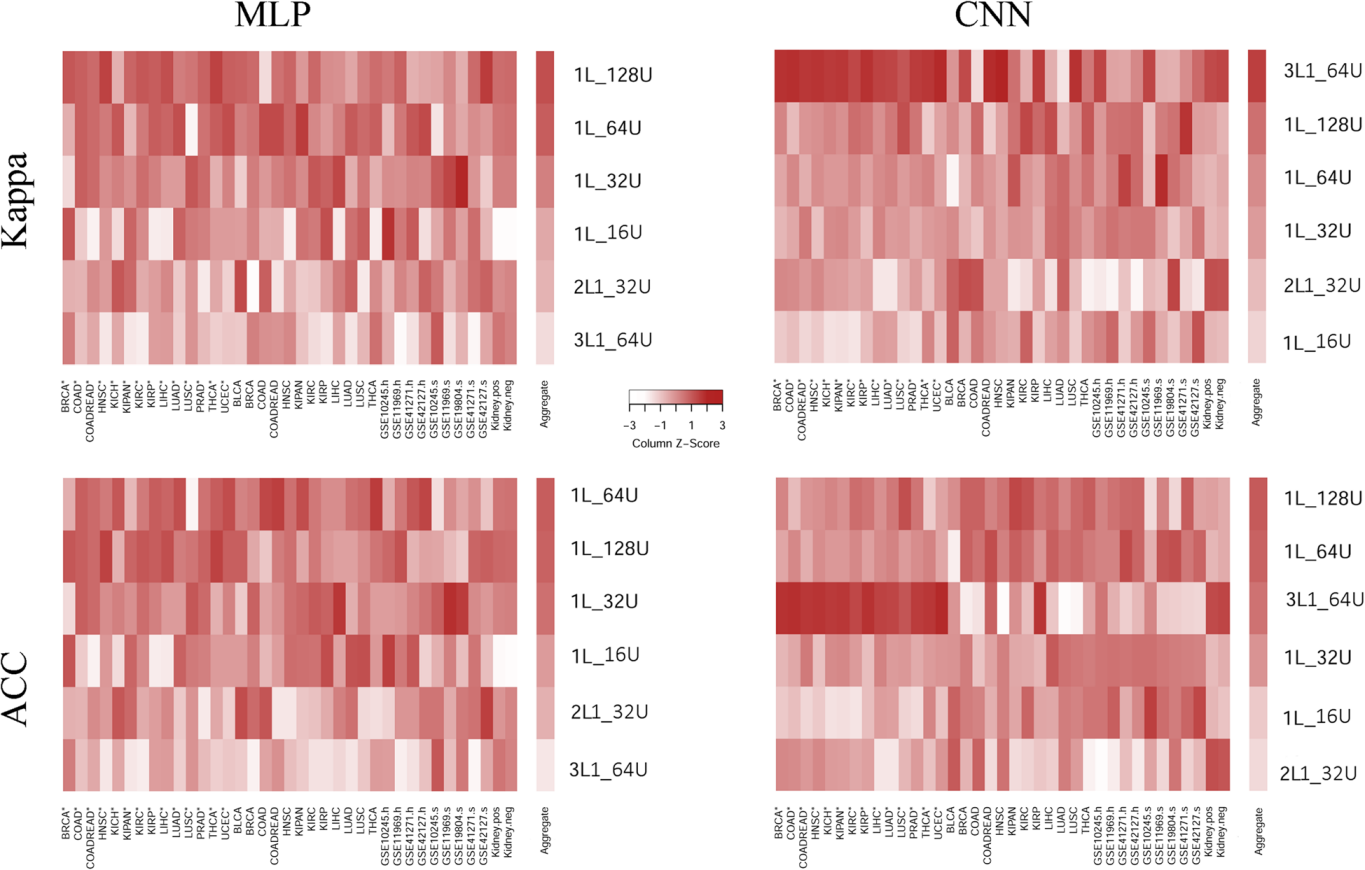
Performance of six architectures of MLP/CNN in classifying 37 datasets. Values in each column were scaled. Architectures were ordered by the mean rank of performance across all 37 datasets (“Aggregate” bar). TCGA transcriptome data were employed for both stage classification (12 cases) and cancer/normal classification (*, 14 cases). Five original NSCLC datasets were organized into nine datasets for stage classification (5 datasets) and histology classification (4 datasets), separately. Two metabolome datasets for chronic kidney disease were adopted to perform classification among 6 classes

The classification performance of six MLP architectures across 37 tasks are visualized in column-scaled heatmaps (Fig. 2, left panes). The Kappa metric clearly indicates that 1L_128U, the single-layered architecture with 128 hidden units, had the best performance. It appears that the performance of single-layered MLPs had a positive dependence on the number of hidden units, as 1L_128U, 1L_64U, 1L_32U, and 1L_16U were ranked as the 1st, 2nd, 3rd, and 4th places in overall Kappa performance (Fig. 2, top-left pane). Precisely, 1L_128U attains the highest Kappa for 16 out of 37 datasets, whereas the other three single-layered MLPs won in fewer datasets (13 times for 1L_64U, 7 times for 1L_32U, and 5 times for 1L_16U). A two-layered MLP with 16/32 units (2L1_32U) and a three-layered MLP with 16/32/64 units (3L1_64U) returned inferior performance than the four single-layered MLPs. The ACC metric gave similar rankings of the six MLP architectures (Fig. 2, bottom-left), although placing the model of 64 units (1L_64U, average rank = 2.71) ahead of the model of 128 units (1L_128U, average rank = 2.78). In terms of ACC, 1L_128U performed best 13 times, and 1L_64U performed best 12 times.

The pattern of CNN performance is not as evident as that of MLP (Fig. 2, right panes). A weak trend can be observed that one single convolutional layer, especially one with a good number of kernels, tends to outperform other architectures. However this trend is violated by the fact that a three-layered structure (3L1_64U) defeated all other architectures in terms of Kappa (Fig. 2, top-right pane). CNN architecture 3L1_64U achieved the best performance in 22 out of the total 37 datasets, including 13 TCGA cancer-vs-normal discriminations, five TCGA stage classifications, two NSCLC classifications, and CKD stage classification using both positive and negative ion metabolomics datasets. In terms of ACC, single-layered CNNs of moderate or large numbers of kernels (128 and 64) outperformed the three-layered CNN model (Fig. 2, bottom-right pane).

A similar composite heatmap figure involving six additional architectures that waived the drop-out design is provided in Additional file 1: Figure S1. Across the expanded architecture set, the phenomenon was still obvious that single-layered MLPs with ample hidden units achieved the best performance, whereas the CNN performance pattern becomes even more obscure. As a side note, we found that MLP models with the drop-out design consistently outperformed MLP models devoid of the drop-out design. This distinction was not apparent with CNN models.

**Fig. 3:**
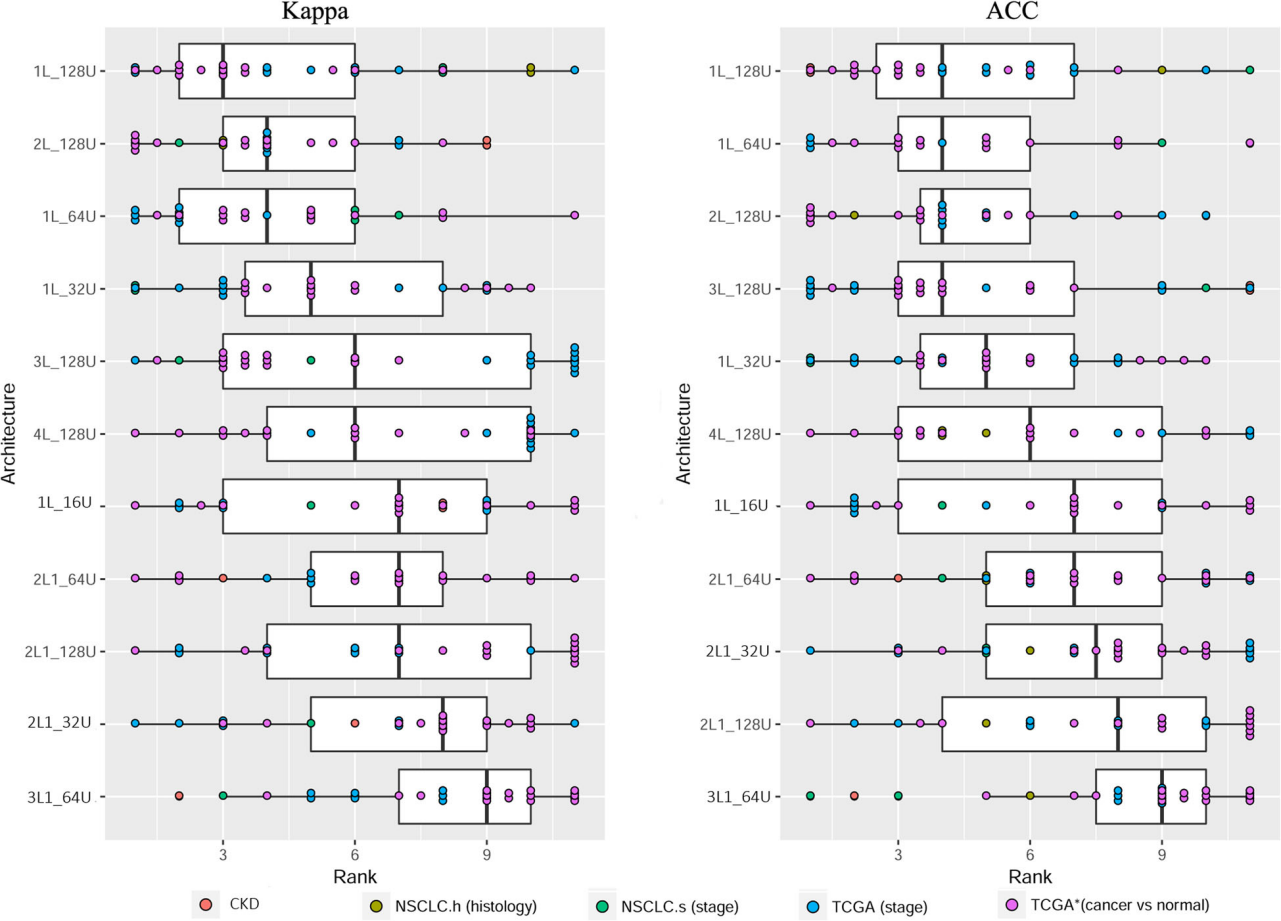
Ranks of classification performance of six primary and five extended architectures of MLP. The original performance measures (Kappa or ACC) were converted to ranks (1–11) within each dataset, with a smaller rank signifying a better performance. Each dot represented a rank per architecture and per dataset. Dots were colored by the dataset group (see section Datasets). Architectures were ordered by the mean rank across all datasets. See Additional file 1: Table S1 for definitions of the various architectures

These results suggest that single-layered MLPs with a moderate-to-great (≥ 64) number of units outperform MLPs of multiple hidden layers. Nevertheless, the six primary architectures covered only one instance of two-layered and one instance of three-layered structures. To fully verify the presumable advantage of single-layered MLPs, we additionally investigated two-layered MLP architectures with alternative unit configurations (2L1_64U and 2L1_128U) and deeper MLPs with equal numbers of units per layer (2L_128U, 3L_128U, and 4L_128U) were tuned to the supposedly optimal value (128). Still, single-layered MLPs 1L_128U and 1L_64U dominated over other architectures, with 1L_128U ranked the best for both Kappa and ACC and 1L_64U ranked the 3rd place for Kappa and the 2nd place for ACC (Fig. 3).

### MLP outperforms CNN and classical machine learning models

For each distinct classification scenario, we determined an optimal architecture for MLP/CNN as the one winning the most datasets and evaluated the performance of MLP and CNN using these optimal architectures. MLP’s optimal architectures were 1L_64U for the scenarios of TCGA (stage classification) and NSCLC (adenocarcinoma vs squamous); 1L_128U for the scenarios of TCGA (cancer vs normal), NSCLC (stage classification), and CKD (stage classification). Of note, 1 L-128 U was not found as the optimal architecture for MLP across all scenarios. 1 L-64 U was found to be nearly as good as 1 L-128 U in the overall evaluation, being voted in the 2nd place by Kappa and in the 1st place by ACC (Fig. 2, left panes). CNN’s best performing architectures were 3L1_64U for all scenarios except for NSCLC (stage classification), which favored 1L_128U.

**Fig. 4:**
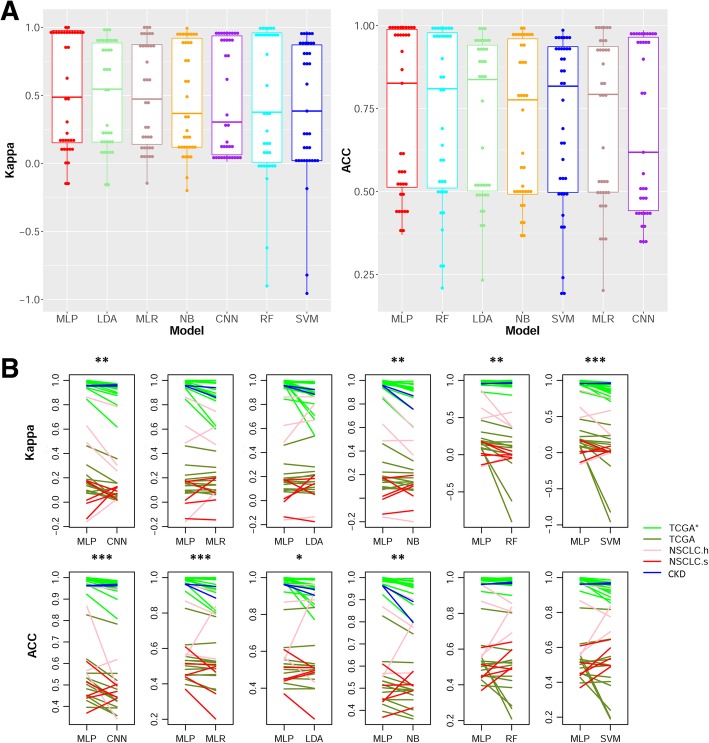
Performance of MLP, CNN, and five classical machine learning models across 37 datasets. **A** boxplots of 37 performance values for each method. From left to right, methods are sorted by descending mean performance. **B** Relative performance comparison between MLP and any one of the other six methods. Each line connects the performance values of two methods on the same dataset. Five colors are used to distinguish the five dataset groups. *p < 0.01. ** *p* < 0.001. ***p < 0.0001

Across all datasets, the average Kappa performance of the seven machine learning models were sorted from best to worst in the following order: MLP, LDA, MLR, NB, CNN, RF, and SVM (Fig. 4a, left). Across all datasets, the average ACC performance of the seven models were sorted from best to worst in the following order: MLP, RF, LDA, NB, SVM, MLR, CNN (Fig. 4a, right). Per either Kappa or ACC, MLP stood out as the best performing method, whereas CNN is not conspicuous in either metric. We further compared MLP against each of the other six methods using a single-tailed Wilcoxon’s Signed Rank Test, finding that MLP significantly outperformed CNN, NB, RF, and SVM in Kappa (all *p*-values < 0.001) and it significantly outperformed CNN, MLR, LDA, and NB in ACC (all p-values < 0.01) (Fig. 4b). Combining the test results for Kappa and ACC, we conclude that MLP defeated each of the six competitor models, no matter whether the competitor is older (MLR, LDA, and NB) or relatively modern (RF, SVM, and CNN).

In addition to demonstrating an overall superiority across all 37 datasets, MLP also achieved statistically significant advantage over most competitors in individual groups of datasets for TCGA (cancer vs normal) and the TCGA (stage classification). For the TCGA (cancer vs normal), MLP outperformed all other models except RF in terms of both Kappa and ACC (*p* < 0.01, Wilcoxon’s Signed Rank Test). For the TCGA (stage classification), MLP outperforms all other models except LDA in either Kappa or ACC (p < 0.01, single-tailed Wilcoxon’s Signed Rank Test). Kappa and ACC for each method and each cancer type unambiguously found MLP as the overall best model for TCGA (cancer vs normal) (Additional file 1: Figure S2). MLP did produce better performance over the majority of the other machine learning methods for the other three test scenarios (NSCLC (adenocarcinoma vs squamous), NSCLC (stage classification), and CKD (stage classification). However, these advantages were not statistically significant. This could be due to limited sample sizes. For example, MLP had higher ACC and Kappa than NB, MLR, LDA, MLP, and SVM in the CKD positive and negative ion metabolomics datasets (Fig. 4b, blue lines), but the advantages cannot be evaluated statistically given that only two datasets were used.

### MLP is robust against imbalanced class composition and inaccurate class labels

**Fig. 5:**
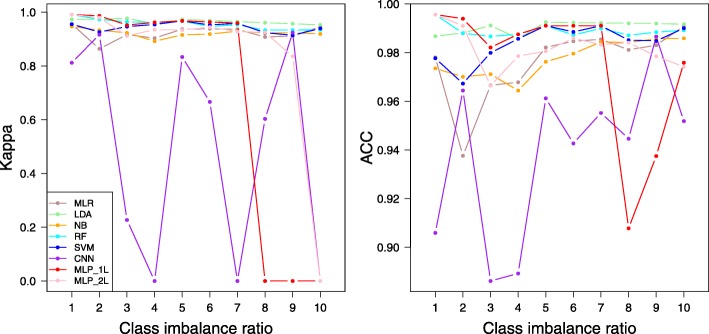
Classification performance over incremental positive-to-negative class ratios. Each data point represents an average value over five repetitive trials. Note that negative Kappa values were truncated at 0. MLP_1L, an MLP architecture of one hidden layer having 128 units. MLP_2L, an MLP architecture of two hidden layers having 128 units on both hidden layers. MLP_1L and MLP_2L precisely map to structures 1L_128U and 2L_128U in Additional file 1: Table S1

Within the TCGA (cancer vs normal) scenario, BRCA (breast cancer) had the largest number of cancer patients and thus was often chosen for elaborated analyses. We performed an investigation of class-imbalance robustness on a series of datasets originating from BRCA. The BRCA dataset contains 112 normal samples and 1093 tumor samples. From this full dataset, we derived a series of 10 datasets with increasing tumor vs normal ratio (112 tumor vs 112 normal, 224 tumor vs 112 normal, … 1090 tumor vs 112 normal). The performance values of each method, in Kappa and ACC, were calculated under each imbalance ratio, and the average performance values of each method were connected to reveal a possible trend over increasing imbalanced ratios (Fig. 5). While all methods had decent ACC values (> 0.88) for all class-imbalance ratios, CNN had excessive Kappa fluctuations across the surveyed range (Fig. 5). Particularly, at imbalance ratios 4 and 7, the average Kappa of CNN dropped to near zero. These absurdly low average Kappa values demonstrated the unstable performance of CNN under class imbalance. For example, the five repetitive datasets at tumor vs normal ratio 4 returned 0.88, 0.25, − 3.04, 0.86, and 0.71 in Kappa values. The unstable Kappa values further proved that CNN is not an ideal machine learning method for classification using numerical matrix-formed omics data.

All classical methods (MLR, LDA, NB, RF, and SVM) maintain excellent Kappa compared to CNN. One-layered MLP had as good Kappa values as the classical methods in a majority of the whole imbalance range, with deterioration to unacceptable levels at the high imbalance end (tumor vs normal ratio ≥ 8). Two-layered MLP, or 2L_128U specifically, had Kappa values > 0.8 until the imbalance ratio elevated to 9, from which the Kappa plunged to negative at the imbalance ratio of 10. Overall, MLP had good robustness against moderate class imbalance, and two-layered MLP showed better robustness than single-layered MLP in this regard.

**Fig. 6:**
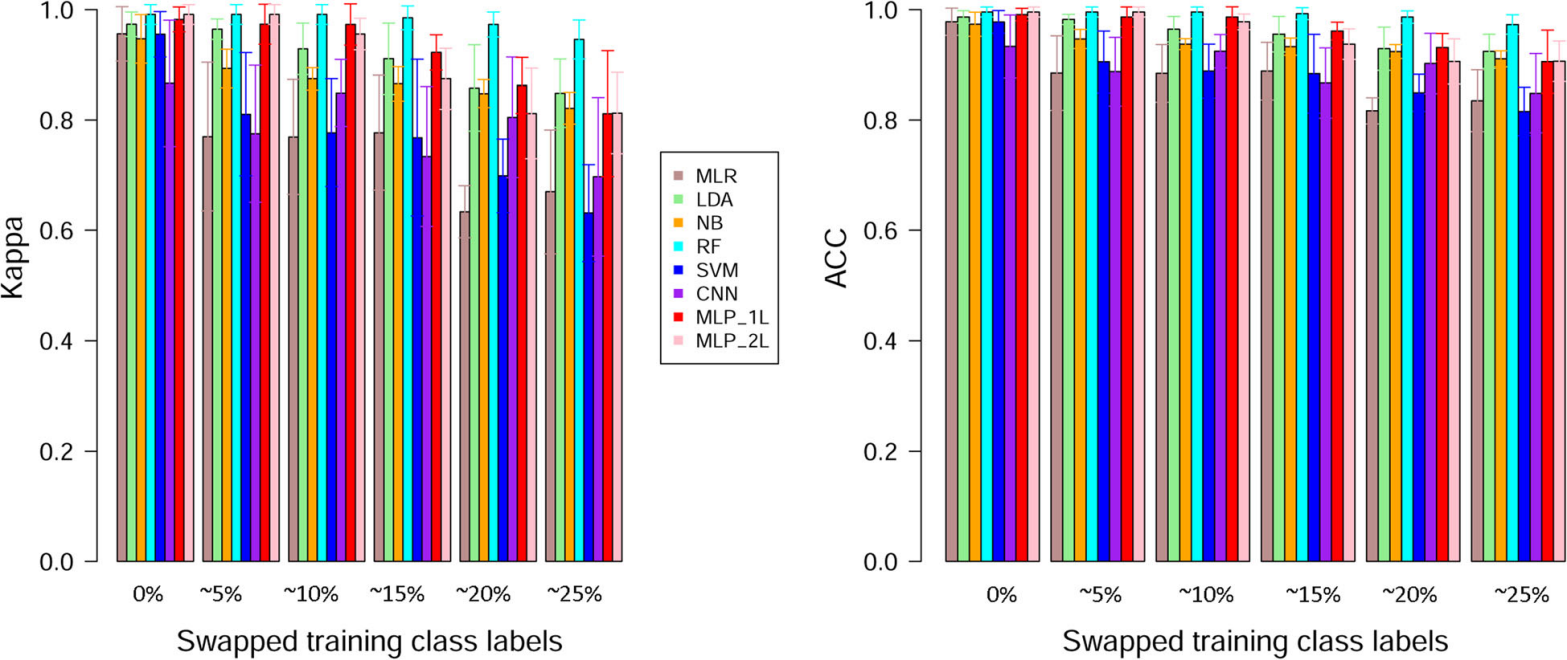
Classification performance over incremental fractions of swapped class labels in training data. MLP_1L and MLP_2L precisely map to structures 1L_128U and 2L_128U in Additional file 1: Table S1

Using a balanced BRCA RNA-seq dataset (with a 1:1 tumor vs normal ratio), we studied the influence of inaccurate class labeling on classification performance. We randomly selected a portion of equal-sized positive examples and negative examples in the training dataset and let them undergo random shuffling of class labels. Note that the class labels of the testing dataset were not altered. The samples undergoing label shuffling accounted for 0, 10, 20, 30, 40, and 50% of the training data, thus generating 0%, ~ 5%, ~ 10%, ~ 15%, ~ 20%, and ~ 25% mislabeled training samples. The mislabeling was repeated three times to generate three repetitive datasets for each inaccuracy level. The performance of each method was summarized across cross-validation datasets and aggregated over the repetitive swapping trials. Grouped barplots present the comparative results in terms of aggregated Kappa and ACC (Fig. 6). As expected, for all methods the classification performance declines as proportion of the mislabeling increased in the training dataset. Surprisingly, even with a quarter of training data points wrongly labeled, all methods preserve good ACC (> 0.81) and acceptable Kappa (> 0.63).

With no mislabeling, RF and MLP-2 L tied as the best performing methods, with exactly the same Kappa of 0.991 and the same ACC of 0.995. As the mislabeling rate increased, RF maintained its high performance. MLP was the second best robustness method. MLP-1 L and MLP-2 L performed neck to neck with slight advantage to MLP-1. LDA followed MLP as the third most robust method.

### CNN and MLP require much greater computation time than traditional models

We empirically evaluated the computation complexity of various machine learning models on the CKD positive ion metabolomics dataset. We derived five cross-validation datasets where each contained a randomly sampled 562 or 563 subjects for training data and the remaining 141 or 140 subjects for testing data. All five classical machine learning models, along with one instance of CNN and four instances of MLP were tested (Table 1). All classical classifiers used a trivial computation time as compared to deep learning models, which consumed tens of thousands times more computation time than the swiftest model LDA. MLP models cost slightly less computation time than CNN. Increasing the depth of MLP models marginally increased the computation time.

**Table 1:** Elapsed computation time (in seconds) for various machine learning models. The positive Kidney dataset was used as the test dataset. All classifiers were implemented on a desktop personal computer with an Intel® Xeon® CPU E5–1650 v4 processor of 3.6 GHz and a random access memory of 32 GB

	Test1	Test2	Test3	Test4	Test5
MLR	0.29	0.27	0.27	0.28	0.28
LDA	0.03	0.01	0.01	0.01	0.02
NB	0.11	0.09	0.09	0.11	0.11
SVM	0.05	0.05	0.05	0.05	0.05
RF	0.64	0.65	0.73	0.65	0.64
CNN_3L	194.19	195.25	196.53	192.28	212.15
MLP_1L	144.70	145.76	144.53	139.73	173.07
MLP_2L	155.72	154.83	154.75	151.88	171.06
MLP_3L	169.61	170.42	178.89	166.91	169.39
MLP_4L	169.83	172.26	179.52	167.61	172.89

## Discussion

Deep learning is finding more and more exciting applications in several domains of bioinformatics [1]. Nevertheless, phenotype classification using numerical matrix-formed omics data has received insufficient attention from deep learning practitioners. Thus, we carried out a survey of various architectures of MLPs and CNNs to pinpoint the most optimal configuration(s) for phenotype classification using RNA-seq and high throughput metabolomics data. Because of the vast computation load necessitated by a large number of tested datasets (Table 2), we had to apply a simple cross-validation schema for assessing seven methods across 93 datasets. In real construction and evaluation of a specific classifier, usually two layers of model validation are required, namely the internal cross-validation and the external independent validation [26]. Secondly, we included both binary classification and multi-class classification problems in our survey, and adopted ACC and Cohen’s Kappa as performance measures because they could handle multi-class problems in the same way as binary problems. The choice of best performance measures is an open question, especially in the multi-class context. Other than Cohen’s Kappa, Matthew’s Correlation Coefficient [26] might be a good measure as well, which could be included as an alternative to Kappa in future evaluation studies.

**Table 2:** Five groups of omics datasets used for testing classification models

Dataset group	Classification problem	# Datasets	# Classes	# Raw features	# Reduced features	# Subjects	Maximum class ratio
TCGA*	TCGA (cancer vs normal)	14x5^a^	2	20,501	40	48–258	1:1
TCGA	TCGA (stage classification)	12	2, 3, or 4	20,501	40	190–974	3:1
NSCLC.h	NSCLC (adenocarcinoma vs squamous)	4	2	21,619 - 54,675	40	58–254	4.3:1
NSCLC.s	NSCLC (stage classification)	5	3	21,619 - 54,675	40	58–265	4.4:1
CKD	CKD (stage classification)	2	6	14,742^b^ and 7,852^c^	54^b^ and 49^c^	703	1.3:1

^a^Five repetitive sets of positive subjects were randomly sampled from the full TCGA samples to match with the negative dataset. Training and testing were performed on each combined dataset, and performance values were averaged across the five repetitive datasets to return one value per cancer type

^b^CKD positive ion metabolomics

^c^CKD negative ion metabolomics

We tested four discrete values for the number of units (neurons) per layer, namely 16, 32, 64, and 128. A positive dependence of classification accuracy on the unit number was observed for MLP. Occasionally, the performance peaked at unit number 64 and plateaued from 64 to 128. In conclusion, when provided with a good number of neurons on the hidden layers, shallow MLPs are sufficient for achieving excellent disease classification accuracy. This conclusion is in agreement with the major finding from a survey of CNN structures for transcription factor binding prediction [22]. While that survey concerned the validity of deep learning models in analyzing DNA sequences, the present study concerns the validity of deep learning models in analyzing numerical matrix-formed data.

CNN is likely to succeed when an intrinsic spatial structure exists within the input data, such as in two-dimensional image processing or one-dimensional DNA/RNA sequence analyses. In our scenario, the features of the omics data matrices do not have an innate spatial structure. We mandated an arbitrary structure among features by convolving the features according to the decreasing order of principal components. Lack of genuine spatial structure within the omics data matrices may primarily account for the failure of CNN. The exploration of CNN in such applications may be expanded to settings that incorporate more reasonable feature structures. For example, genes can be grouped to functional terms through Gene Ontology, or they can be organized into clusters according to expression similarity. Indeed, custom CNN models with these very innovative features have appeared in preprint manuscripts [18, 19]. Despite the possibility of improvement, our present work surveyed a representative range for major architecture parameters, namely network depth and width, and we did not detect any promising signal from our numerous trials. Our results tend to resonate with a negative perspective into CNN application in well structured genomics data [23]. At least, our results indicate that a CNN with fine-tuned depth or width is still unlikely to overshadow conventional competitors. Substantial innovation and meticulous benchmarking is needed before CNN can establish a promising role in omics data analysis, especially when dealing with transcriptome data.

## Conclusions

In conclusion, we found that single-layered MLPs (i.e., MLPs of one hidden layer), and occasionally two-layered MLPs, achieved the best classification performance as long as they were deployed with ample neurons on the hidden layers. The performance of CNN classifiers was inferior compared to MLP, and no evident pattern can be discerned for CNN. Furthermore, when compared with classical machine learning method such as RF and SMV, well-configured MLPs demonstrated an overall best performance. In the face of suboptimal class composition and mislabeled training data, single-layered or two-layered MLPs retained satisfactory robustness. In summary, our results proved that single-layered or two-layered MLP models are a good choice for performing phenotype classification on matrix-formed omics data. The results also dispelled the anticipation of excellent performance of CNN in such scenarios. Although slower compared to classical machine learning methods, the extra computation time used by MLP is still tolerable. The only scenario in which we do not recommend using MLP is for extremely imbalanced classification datasets.

## Methods

### Datasets

In this study, we obtained 37 high-throughput omics datasets from three sources, and organized them into five groups (Table 2). First, RNA-seq data in RSEM format were downloaded from The Cancer Genome Atlas (TCGA) via the R package TCGA2STAT [27] and were log transformed. Cancer stages with number of subjects less than 50 were discarded, and cancer types with two or more eligible stages were retained. As a result, 12 TCGA cancer types (COAD, KIRP, COADREAD, KIPAN, KIRC, THCA, BLCA, BRCA, HNSC, LIHC, LUAD, and LUSC) were used for supervised classification of cancer stages. Cancers COAD and KIRP were divided into two stages; cancers COADREAD, KIPAN, KIRC, and THCA were divided into four stages; all other six cancers, namely BLCA, BRCA, HNSC, LIHC, LUAD, and LUSC, were divided into three stages. From a different perspective, we derived binary-class datasets from 14 TCGA cancers (BRCA, COAD, COADREAD, HNSC, KICH, KIPAN, KIRC, KIRP, LIHC, LUAD, LUSC, PRAD, THCA, and UCEC), focusing on the tumor vs. normal distinction. Since normal samples always account for a minor portion in the TCGA data cohort, we randomly sampled an equal number of tumor samples to match with the normal samples of each cancer type. The random subsampling process was repeated five times, and model training and testing were separately performed on each dataset and averaged across the five trials to return a summary evaluation per cancer type.

Second, we downloaded five RNA-seq datasets for multiple stages of non-small cell lung cancer (NSCLC) patients from Gene Expression Omnibus. These five datasets were accessed via IDs GSE10245, GSE11969, GSE19804, GSE41271, and GSE42127, which involved 58, 144, 59, 265, and 174 human subjects, respectively. All subjects from these five datasets were NSCLC stage I, II, or III patients. Except for GSE19804, all other four datasets were reorganized for binary histology classification between adenocarcinoma and squamous NSCLC.

The last two dataset used were high throughput metabolomics datasets generated from 703 subjects in a chronical kidney disease (CKD) study. The 703 subjects include 587 CKD patients with five stages (CKD1 = 120, CKD2 = 104, CKD3 = 110, CKD4 = 119, CKD5 = 134) and 116 age-matched normal healthy controls. The metabolomics dataset were generated by ultra-performance liquid chromatography-high-definition mass spectrometry in both positive and negative ion modes, respectively. The metabolomics data were properly normalized following established guideline [28].

In summary, 37 datasets were collected for this study. Each dataset comprised tens of thousands of molecules, so a beforehand feature reduction was unavoidable. To be consistent with a companion ongoing project, we used LASSO [29] to handle the two CKD datasets, and as a result, retained 54 and 49 variables for the CKD-positive and CKD-negative datasets, respectively. Because LASSO did not work on some of the other datasets (i.e., LASSO did not reduce the number of features), we applied the commonly used Principal Component Analysis to select a comparable number of features, namely, 40. Although nowadays computation resource is not as a limiting factor as in the past, our study involved repeated model training/testing on a large number of datasets each involving tens of thousands of raw features (Table 2), which would pose extreme computation burden if without any feature reduction. LASSO and Principal Component Analysis effectively reduced the number of features of these datasets to a manageable scale suitable for our comparative evaluation purpose. Feature reduction operation was practiced in another deep learning study [24] as well.

### Deep learning models

We examined two major variants of modern neural networks, Multi-Layer Perceptrons (MLP) and Convolutional Neural Networks (CNN), for our survey. MLPs encompass two-layered feed-forward neural networks which had proven validity in the supervised classification of microarray data. We formalized various architectures of MLPs, making sure to cover deeper models with more than one hidden layer. Recently, CNNs have been successfully employed in sequence analyses of DNA/RNA data [2], and efforts to exploit CNN in numerical omics are on the rise [18, 19]. It’s worthwhile to to investigate if CNN proves as promising in numerical classification as in image recognition at the onset of the trend.

The activation functions for all layers except the output layer were always Rectified Linear Units [30], while the output layer used the softmax activation function [31]. The “rmsprop” optimizer [32] and the “categorical_crossentropy” loss function [33] were chosen for all MLP and CNN models. Within each fold of the outer 5-fold cross validation, an inner 5-fold partition of the training dataset was imposed and the inner loop of 5-fold cross validations was utilized to choose the optimal batch size of training examples, from three candidate values (1, 32, and the number of all training examples). For the inner cross-validation implementations, MLP and CNN were configured to learn 1000 epochs of the training datasets, however after completing the inner cross validations the minimal epochs achieving the best validation accuracy were recorded for each value of the batch_size parameter. For the outer cross-validation implementations, the batch size was set as the dataset-specific optimal value and of the optimal epochs (< 1000) determined from the inner cross validations was exerted for saving unnecessary computation time. In most cases, we found the optimized batch size was one.

We largely followed a previous study by Zeng et al. [22] to devise basic architectures of MLP and CNN models (Fig. 1), from which variant forms with more units per layer and/or more layers were derived (Additional file 1: Table S1). Of note, for narrative ease, we encode the various architectures according to the number of hidden layers, without counting the output layer. For instance, our basic single-layered architecture 1L_16U (Fig. 1) would be called a two-layered network in common terminology. We tested different network widths by setting the number of units at 16, 32, 64, or 124 (Additional file 1: Table S1). These values accorded with the parameter setting in Zeng et al.’s work [22] yet bearing an additional interpolated value (32) to ensure a more even coverage of the tested range. We also investigated the effect of dropping-out [34], a novel technique proposed to mitigate overfitting. By incorporating or removing a final dropout layer which randomly drops out a half of input units) 6 × 2 architectures for both MLP and CNN were formed (Additional file 1: Table S1), upon which we explored possible width-dependent and/or depth-dependent performance patterns. Afterwards, we extended our survey to additional deep MLP architectures containing 2, 3, or 4 hidden layers (Additional file 1: Table S1), arriving at a more definite conclusion on optimal architecture(s) of MLP and CNN for supervised classification using matrix-formed omics data. All deep learning functionalities were offered by the R package keras [35].

### Classical machine learning models

We examined five classical machine learning models to compare with MLP and CNN. Linear Discriminant Analysis (LDA) realizes classification by seeking a linear combination of features that best separate the labeled objects. The R package MASS [36] was utilized for LDA computation. Multinomial logistic regression (MLR) is an extension of binomial logistic regression to allow for a dependent variable with more than two categories. In essence, MLR models the logit transformation of probability-wise class membership through a linear summation of various dependent variables. The R package nnet [36] was utilized for MLR computation. Naïve Bayes (NB) is a probabilistic classifier that applies Bayes' theorem with strong (naive) independence assumptions between the features. Although the assumption of NB usually does not hold in practical applications, NB often yields acceptable classification performance and may outperform sophisticated algorithms. The R package klaR [37] was utilized for NB computation, where any feature with zero variance in a class was dropped beforehand.

Random Forest (RF) is an ensemble model performed by constructing a multitude of decision trees at training time and outputting the class that is the mode of the classes. Since its introduction in the 1990s [38], RF has been widely applied in biomedical applications with great successes. The R package randomForest [39] was utilized for RF computation, where the number of trees was set at the suggested value 500.

A Support Vector Machine (SVM) is a representation of the examples as points in space, mapped so that the examples of the separate categories are divided by a clear gap that is as wide as possible. SVM was invented in the 1960s and significantly enhanced in the 1990s [40]. The R package e1071 [41] was utilized for SVM computation, where a linear kernel was adopted.

### Classification performance measures

We used two metrics to measure the performance of each method. All aforementioned models provide classification output for each test example as a score vector, which are the probabilities of assigning the examples to each class. Such a score vector is transformed to a class designation by selecting the class with the highest score. Classification Accuracy (ACC) is defined as the proportion of samples that are correctly classified to their class labels. Cohen’s Kappa statistic [42] is an effective but under-utilized metric that has a special advantage for machine learning cases involving multiple classes and/or imbalanced class distribution. More details regarding Kappa statistics are presented in the Additional file 1: Table S2. Most analyses conducted in this study used five-fold cross validation, with four-fold cross validation applied to only GSE10245 and GSE19804, the datasets with limited number of samples (< 60).

## Additional file

Additional file 1: Supplementary text, table, and figures. Here we included explanation to Cohen’s Kappa, **Table S1**, **Table S2**, and **Figures S1** & **Figure S2** cited in the manuscript. (PDF 807 kb)

## Abbreviations

ACC: Accuracy
BLCA: Bladder urothelial carcinoma
BRCA: Breast invasive carcinoma
CKD: Chronic kidney disease
CNN: Convolutional Neural Network
COAD: Colon adenocarcinoma
COADREAD: Colorectal adenocarcinoma
HNSC: Head and Neck squamous cell carcinoma
KICH: Kidney Chromophobe
KIPAN: Pan-kidney cohort (KICH+KIRC+KIRP)
KIRC: Kidney renal clear cell carcinoma
KIRP: Kidney renal papillary cell carcinoma
LDA: Linear Discriminant Analysis
LIHC: Liver hepatocellular carcinoma
LUAD: Lung adenocarcinoma
LUSC: Lung squamous cell carcinoma
MLP: Multi-Layer Perceptron
MLR: Multinomial Logistic Regression
NB: Naïve Bayes
NSCLC: Non-small cell lung cancer
PRAD: Prostate adenocarcinoma
RF: Random Forest
SVM: Support Vector Machine
TCGA: The Cancer Genome Atlas
THCA: Thyroid carcinoma
UCEC: Uterine Corpus Endometrial Carcinoma
