# Supplementary material for: Architectures and accuracy of artificial neural network for disease classification from omics data
Source: BMC Genomics. 2019 Mar 4;20:167. doi: 10.1186/s12864-019-5546-z (PMC6399893; doi:10.1186/s12864-019-5546-z)
Supplement: Supplementary file 1 — Supplementary text, table, and figures. Here we included explanation to Cohen’s Kappa, Table S1, Table S2, and Figures S1 & Figure S2 cited in the manuscript. (PDF 807 kb) [file 12864_2019_5546_MOESM1_ESM.pdf]

Architectures and accuracy of artificial neural network for disease classification from omics data

Hui Yu[1], David C. Samuels[2], Ying-yong Zhao[3], Yan Guo[1,§]

§correspondence: yaguo@salud.unm.edu

Additional file 1
File name: Additional file 1.pdf

Title of data: Supplementary text, table, and figures to the manuscript.

Description of data: Here we included Table S1,Table S2 (explanation to Cohen's Kappa), and Figures S1 & S2 cited in the manuscript.

Table S1. The code and a brief description of 11 variants of MLP/CNN models surveyed in this work. The top six structures are primary ones used for making initial observations; the bottom five ones are extended structures used to verify initial speculations. Unless explicitly denied, all networks contained a dropout layer immediately prior to the output layer with a dropping rate of 0.5. The same code nomenclature was applied to both MLP and CNN models, although in CNN a hidden layer is actually a convolution layer and a unit is more often termed a kernel.

**Simple code**	**Full code**	**Architecture**
1L_16U	1L_16U	The basic structure as depicted in Figure 1.
1L_32U	1L_32U	Use 32 nodes on the first hidden layer.
1L_64U	1L_64U	Use 64 nodes on the first hidden layer.
1L_128U	1L_128U	Use 128 nodes on the first hidden layer.
2L1_32U	2L_16U_32U	Two hidden layers with 16/32 units.
3L1_64U	3L_16U_32U_64U	Three hidden layers with 16/32/64 units.
1L_16U_noDrop	1L_16U_noDrop	Same as 1L_16U except lacking the dropout layer
1L_32U_noDrop	1L_32U_noDrop	Same as 1L_32U except lacking the dropout layer
1L_64U_noDrop	1L_64U_noDrop	Same as 1L_64U except lacking the dropout layer
1L_128U_noDrop	1L_128U_noDrop	Same as 1L_128U except lacking the dropout layer
2L1_32U_noDrop	2L_16U_32U_noDrop	Same as 1L_32U except lacking the dropout layer
3L1_64U_noDrop	3L_16U_32U_64U_noDrop	Same as 1L_64U except lacking the dropout layer
2L1_64U	2L_16U_64U	Two hidden layers with 16/64 units.
2L1_128U	2L_16U_128U	Two hidden layers with 16/128 units.
2L_128U	2L_128U_128U	Two hidden layers with 128/128 units.
3L_128U	3L_128U_128U_128U	Three hidden layers with 128/128/128 units.
4L_128U	4L_128U_128U_128U_128U	Four hidden layers with 128/128/128/128 units.

Cohen's Kappa statistic (Cohen, 1960) is a very useful but under-utilized metric that has a special advantage for machine learning cases involving multiple classes and/or imbalanced class distribution. Suppose it is a k-class problem, where the a priori class distribution is represented in a frequency vector (p_1, p_2, …, p_i, …, p_k), and the predicted class distribution appears as (q_1, q_2, … q_i, …, q_k). For instance, in a three-class classification setting (Table S1), the actual class frequencies are (p_1, p_2, p_3), and the predicted class frequencies are (q_1, q_2, q_3), where $p_i=(n_{i1}+n_{i2}+n_{i3})/N$ and $q_i=(n_{1i}+n_{2i}+n_{3i})/N$ with $N=n_{11}+n_{12}+n_{13}+n_{21}+n_{22}+n_{23}+n_{31}+n_{32}+n_{33}$. The Kappa statistics is defined as *(ACC-expACC)/(1-expACC)*, where $expACC=\Sigma(p_i*q_i)$ and ACC is Accuracy defined above. With regards to Table S1, $expACC=p_1 \cdot q_1+p_2 \cdot q_2+p_3 \cdot q_3$ and $ACC=(n_{11}+n_{22}+n_{33})/N$. Note that this definition of expected accuracy (expACC) is a more realistic approximation of random guess accuracy, which is equal to or larger than the class-distribution-agnostic baseline (1/k).

Table S2 Setting-up for Kappa calculation in a conceptual three-class classification problem.

		**Predicted membership**			**Actual class frequency**
		C1'	*C2'*	*C3'*	
**Actual membership**	*C1*	n_{11}	n_{12}	n_{13}	p_1
	C2	n_{21}	n_{22}	n_{23}	p_2
	C3	n_{31}	n_{32}	n_{33}	p_3
**Predicted Class frequency**		q_1	q_2	q_3	N/A

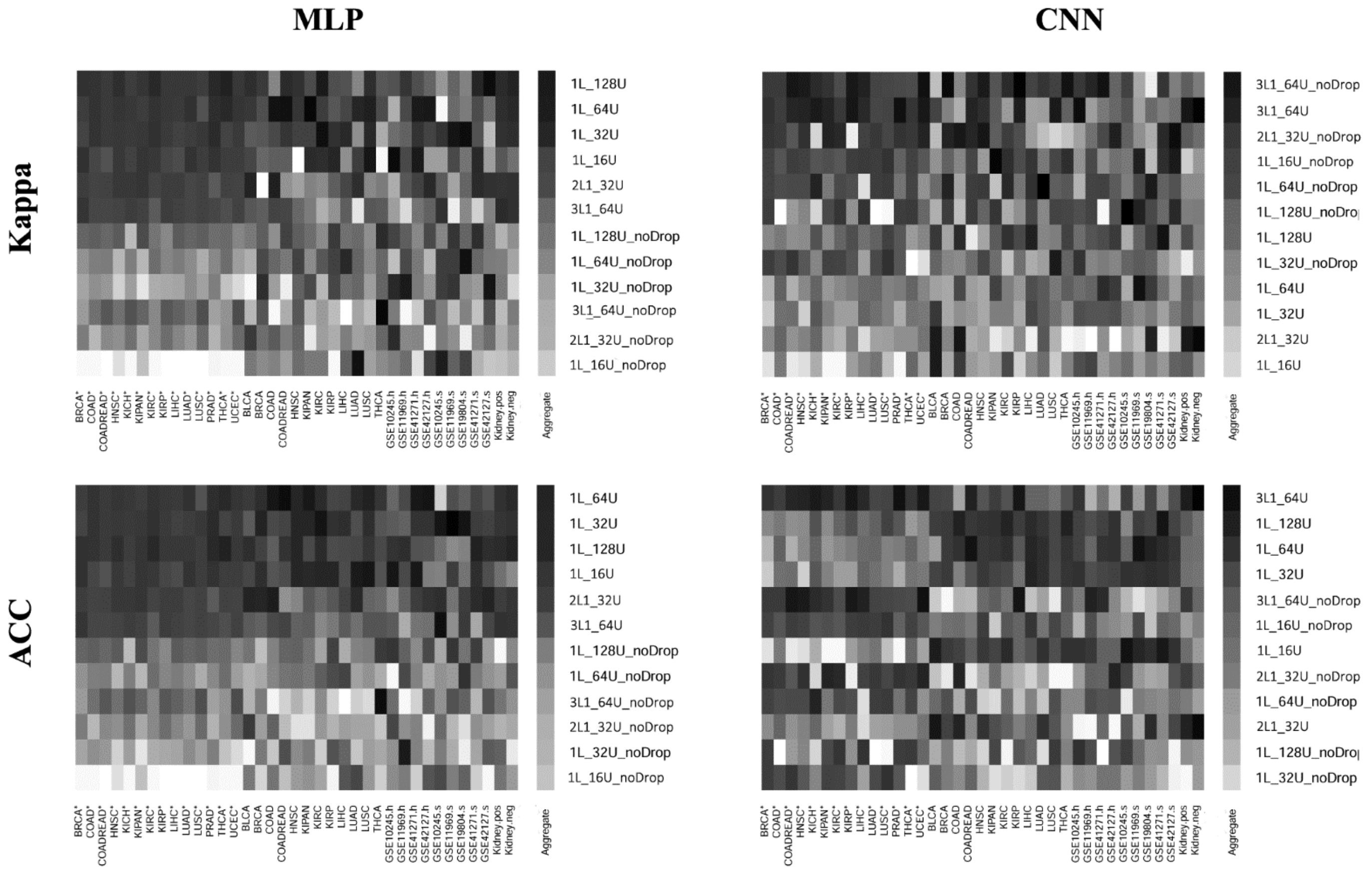

Figure S1. Performance of twelve architectures of MLP/CNN in classifying 37 datasets (TCGA cancers, NSCLC cancers, and kidney disease). The twelve architectures comprise two analogous sets, one with the final dropout layer and the other without (suffixed with “noDrop”). See Table 1 for formal definitions of these architecture codes. TCGA transcriptome data were employed for both stage classification (12 cases) and cancer/normal classification (*, 14 cases). Five original NSCLC datasets were organized into nine datasets for stage classification (5 datasets) and histology classification (4 datasets), separately. Two metabolome datasets for chronic kidney disease were adopted to perform classification among 6 classes.

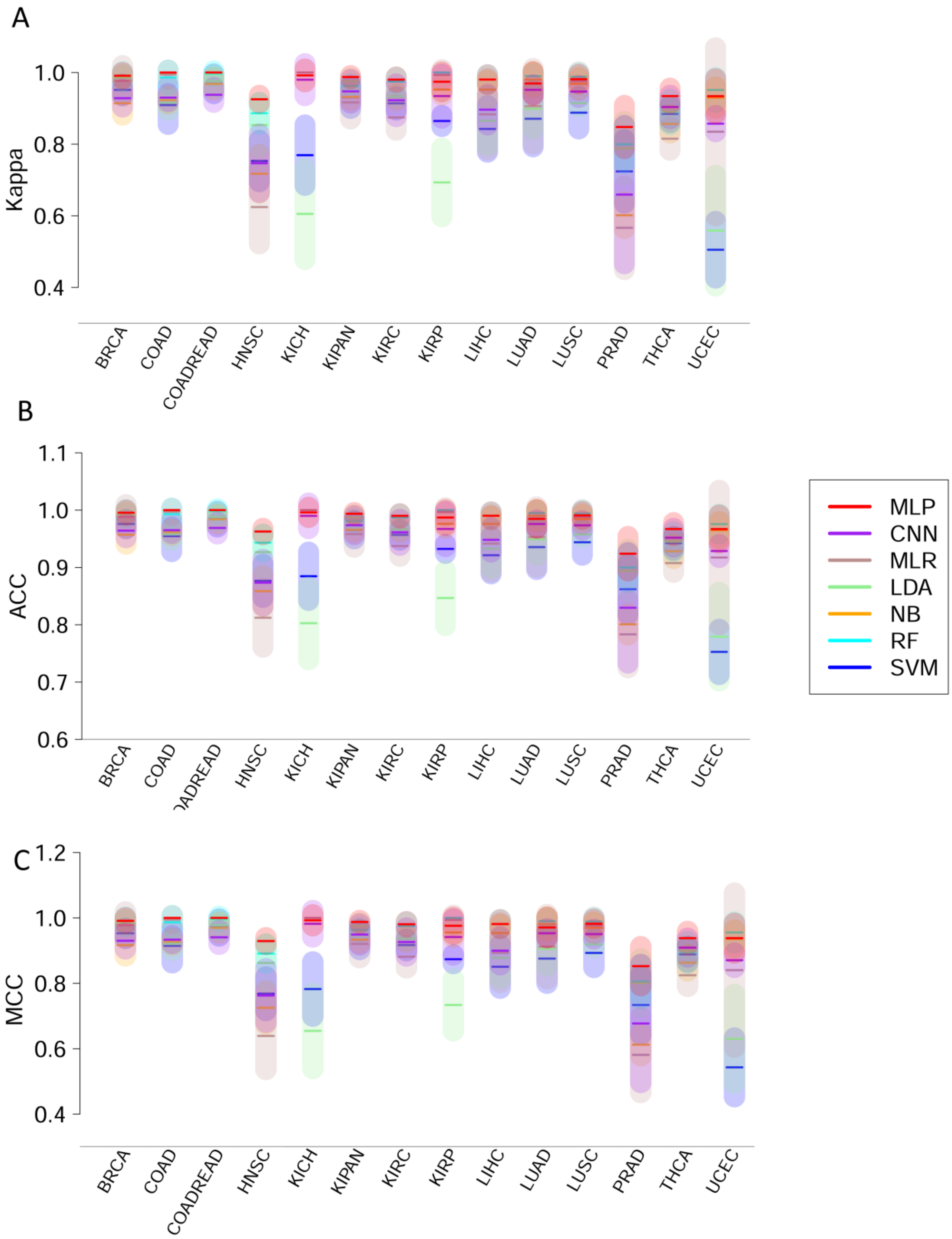

Figure S2. Performance of MLP, CNN, and five state-of-the-art machine learning models in classifying tumor and normal samples of 14 TCGA cancers. At each cancer, the performance of each method is shown with a mean level and a standard deviation interval, which result from five repetitive datasets. A, Cohen's Kappa. B, Accuracy. C, Matthew's Correlation Coefficient.
